# Physiological Action of Baryta and Oxalic Acid

**Published:** 1864-02

**Authors:** 


*Physiological Action of Baryta and Oxalic Acid.*—Dr. Onsum has communicated his researches in the toxical action of the Salts of Baryta and those of Oxalic Acid in *Virchow's Archiv.*, (vol. 28). From these he follows that they form the insoluble precipitates of Sulphate of Baryta and Oxalate of Lime, when they come in contact in the blood with a sulphate or a salt of lime, and that these precipitates obstruct the branches of the pulmonary arteries. Chemical analysis has, in all instances, proved their presence in the lungs, while they could not be discovered either in the brain, the spinal marrow, the kidneys, or the muscles.—*Druggists' Circular.*

